# Discovery of Endothelium and Mesenchymal Properties of Primo Vessels in the Mesentery

**DOI:** 10.1155/2013/205951

**Published:** 2013-04-15

**Authors:** An Ping, Su Zhendong, Dai Jingxing, Liu Yaling, Kyung-Hee Bae, Tan Shiyun, Luo Hesheng, Kwang-Sup Soh, Yeon Hee Ryu, Sungchul Kim

**Affiliations:** ^1^Renmin Hospital of Wuhan University, Wuhan 430-060, China; ^2^Chinese Traditional Veterinary Laboratory, Department of Animal Medicine, Northeast Agriculture University, Harbin 150-030, China; ^3^Department of Human Anatomy, Southern Medical University, Guangzhou 510-515, China; ^4^Nano Primo Research Center, Advanced Institutes of Convergence Technology, Seoul National University, Suwon 443-270, Republic of Korea; ^5^Department of Acupuncture, Korea Institute of Oriental Medicine, Daejeon 305-811, Republic of Korea; ^6^Center of Amyotrophic Lateral Sclerosis, Wonkwang University Hospital, Gwangju 503-310, Republic of Korea

## Abstract

Recent evidences demonstrated that endothelial-to-mesenchymal transition (EndMT) has a crucial role in cancer and is recognized as a unique source of cancer-associated fibroblasts (CAFs). Primo vascular system (PVS) is a new circulatory system which may play an important role in cancer metastasis and regeneration. In the current study, we applied previously established time-saving method to identify primo vessels and further investigated the immunocytochemical properties of primo vessels. Both primo vessels and primary primo vessel cells in the mesentery expressed endothelial markers and fibroblast markers. Double-labeling experiments demonstrated that endothelial and fibroblast markers are coexpressed in primo vessels. In addition, under the stimulation of TGF-*β*1 *in vitro,* primary primo vessel cells differentiated into fibroblasts. Therefore, we found that primo vessels in the mesentery had a transitional structure between endothelium and mesenchymal. This is a new finding of EndMT in normal postnatal animals.

## 1. Introduction

Recently, growing evidence manifested that endothelial cells could be triggered to acquire a mesenchymal phenotype (fibroblast-like cells and loss of cell-cell contacts), obtain mesenchymal biological properties (invasive and migratory capabilities), and gain mesenchymal markers [1–4]. These studies indicated that there existed a unique population of cells that coexpressed both endothelial marker CD31 (also known as platelet endothelial cell adhesion molecule-1 (PECAM-1)) and fibroblast markers, such as PDI, MTS1 (S100A4, also known as FSP1), and procollagen I. Researchers supposed that these recruit fibroblasts mainly arose from endothelial cells and categorized this process as endothelial-to-mesenchymal transition (EndMT). EndMT was first detected in embryonic heart development and later was observed in a variety of pathologic procedures, including cancer, chronic kidney fibrosis, cardiac fibrosis, chronic pulmonary hypertension, atherosclerosis, and wound healing [4–13]. Although the function and precise molecular mechanisms of EndMT in some postnatal diseases have not yet been determined, their determinant role in cancer was conclusively established. Now EndMT was recognized as a crucial source of cancer-associated fibroblasts (CAFs) which modulated cancer progression and played a definitive role in cancer metastasis, microenvironment and angiogenesis [4, 12, 14–21]. EndMT was a critical process and mechanism contributed to a setting of pathological diseases [5, 11, 22]. Does EndMT also occur in normal postnatal states? Till now, no answer can be found.

Primo Vascular System (PVS) is a new kind of circulatory system beyond vascular and lymphatic system [23]. On International Symposium of Primo Vascular System 2010 (ISPS2010), the functional role of PVS in cancer, especially in tumor metastasis, and regeneration was extensively discussed [24, 25]. In this work, we investigated cellular properties of the PVS in the mesentery with immunocytochemistry by studying the primo vessels and their primary cultured cells with endothelial and mesenchymal antibodies. In previous studies [23], there were much work with DAPI and phalloidin to establish the histological characteristics of PVS which we repeated in this work for searching and identifying the PVS in the mesentery specimens. Because thorough immunocytochemical study with the endothelial antibodies and mesenchymal antibodies to characterize the primo vessel was lacking, the EndMT was not noticed before. Furthermore, no study was ever performed with primary cultured cells of the PVS. By performing this study, we found not only the immunocytochemical properties of endothelial and mesenchymal cells of the PVS in the mesentery but also a novel case of EndMT in a normal postnatal animal.

Our study showed that primo vessels in the mesentery coexpressed endothelial markers (CD31, VE-cadherin, vWF) along with fibroblast markers (PDI, MTS1, procollagen I). Primary primo vessel cells also coexpressed these markers and showed a fibroblast-like phenotype. Further investigation revealed that over 14 days culture *in vitro*, primo vessel cells differentiated into fibroblasts and showed increased levels of collagen *α*1(I) mRNA and protein secretion. We deduced primo vessels in the mesentery as a unique structure consisting of EndMT cells. To our knowledge, this might be the first evidence to validate a transitional structure between endothelium and mesenchymal in normal postnatal animals. We believe that this discovery will enrich the recognition and concept of EndMT which maybe exist in normal tissues and conductive to angiogenesis.

## 2. Materials and Methods

### 2.1. Animals

Seven-week-old male and female Sprague-Dawley rats, weighing 200–250 g (Jung-Ang Laboratory Animal Company, Seoul, Korea), were used in this study. For each separate experiment, three rats of each gender were used. Animal care, maintenance, and surgery were performed in accordance with the international laws and policies of the Care and Use of Laboratory Animals, National Academy Press (1996).

### 2.2. Sample Preparation

Rats were anaesthetized with urethane (1.5 g/kg) (Sigma, MO) i.m., and then the abdomens were opened. Under a stereomicroscope (SZX12, Olympus, Japan), primo vessels in the mesentery between the colon and the root of mesentery and between the colon and the small intestine were carefully detected. Primo vessels with a characteristic appearance of semitransparent, white thread-like lines were taken out and were carefully checked under the stereomicroscope to avoid the involvement of vascular vessels and lymphatic vessels. For immunofluorescent study, vascular vessels and lymphatic vessels were chosen as controls. After embedding in OCT (Sakura Finetek, CA), all the tissues were rapidly frozen over liquid nitrogen and stored at −80°C until processed.

### 2.3. Phalloidin and DAPI Staining in Tissue Samples and Cryosections

To confirm the role of phalloidin combined with 4′,6-diamidino-2-phenylindole (DAPI) staining in distinguishing primo vessels from vascular vessels and lymphatic vessels, we performed phalloidin (Invitrogen, CA) and DAPI (Invitrogen, CA) staining in tissue samples and cryosections at first. The main procedure was as follows. Fresh tissues or 10 *μ*m-thickness cryosections were fixed in 3.7% formaldehyde at room temperature (RT) for 10 min. After being washed with 0.1 M phosphate buffered saline (PBS) three times, the samples were further treated with 0.2% Triton-X100 for 5 min. Then, the tissues were washed in PBS and incubated with Alexa Fluor 568 phalloidin (1 : 50) for 2 h in the dark at room temperature (RT). After PBS washing, DAPI (0.1 mg/mL) was added and incubated for 30 min, and then tissues were mounted with prolong gold antifade reagent (Invitrogen, CA). Staining was analyzed independently by two investigators using a fluorescence microscope (BX51, Olympus, Japan).

### 2.4. Isolation of Primary Primo Vessel Cells and Cell Culture

Primary primo vessel cells were obtained from 7-week-old SD rats according to the following procedures. Briefly, primo vessels in the mesentery were gently washed three times with PBS, moved into a cell culture dish, and minced with a disposable surgery knife for approximately 2 min. Tissues were incubated in a solution of 1% collagenase type I (Sigma, MO) dissolved in Hank's balanced salt solution at 37°C for 2 h. Then, the suspension was centrifuged (1200 g for 3 min), and the cells were seeded in a six-well cell culture plate at the desired density (2.5 to 5 × 10^5^ cells per well) and cultured in Dulbecco's modified Eagle's medium (DMEM, Gibco by Invitrogen, CA) supplemented with 10% fatal bovine serum (FBS, Gibco by Invitrogen, CA), 100 U/mL of penicillin (Gibco by Invitrogen, CA), and 100 *μ*g/mL of streptomycin sulfate (Gibco by Invitrogen, CA) at 37°C in a 5% CO_2_ incubation. After 30 min of incubation, suspension cells were removed, and adherent primo vessel cells were cultured in the above medium. Cell cultures were observed by phase contrast microscopy to verify growth, and viability was routinely checked by a trypan blue exclusion assay (Sigma, MO). Cultures showing viability over 95% were used. Culture medium was replaced every 3 d.

The murine fibroblast NIH3T3 cell line and the rat fibroblast BHK-21 cell line were chosen as controls in this study. They were obtained from SNU cell bank (Seoul, Korea) and were cultured in DMEM supplemented with 10% FBS, 100 U/mL of penicillin, and 100 *μ*g/mL of streptomycin sulfate. 

### 2.5. Immunohistochemistry and Phalloidin Staining

Tissue samples were cut into 10 *μ*m longitudinal cryosections for immunofluorescence staining. In single staining, sections of primo vessels were rinsed in PBS and fixed in 4% PFA at 4°C for 10 min. After washing (3 × 10 min in PBS), fixed sections were permeabilized with 0.1% Triton X-100 in PBS for 15 min. Nonspecific binding was blocked by incubation with serum solution for 2 h at room temperature. Then, sections were incubated with primary antibodies overnight at 4°C at the following dilutions: monoclonal anti-CD31 antibody (mouse anti-rat) at 1 : 200, polyclonal anti-vWF antibody (rabbit anti-rat) at 1 : 200, monoclonal anti-PDI antibody (mouse anti-rat) at 1 : 400, polyclonal anti-FSP1 antibody (rabbit anti-rat) at 1 : 200, polyclonal anti-VE-cadherin antibody (rabbit anti-rat) at 1 : 200, polyclonal anti-procollagen I antibody (rabbit anti-rat) at 1 : 400, and polyclonal rabbit anti-LYVE-1 (rabbit anti-rat) at 1 : 200 (Santa Cruz Biotechnology, CA). To detect immunofluorescence, sections were washed and incubated with Alexa Fluor 488- (1 : 400) or 594- (1 : 400), (Invitrogen, CA) for 2 h in the dark at RT. After washing in PBS, sections were incubated with Alexa Fluor 488 phalloidin or Alexa Fluor 568 phalloidin (1 : 50) for 2 h. Counterstaining was performed with DAPI, and the glass coverslips were mounted on the slides with prolong gold antifade reagent. As negative controls, the primary antibody was replaced with nonimmune IgG. Staining was analyzed independently by two investigators using a fluorescence microscope and further processed with Adobe Photoshop CS5 extended computer software.

### 2.6. Immunocytochemistry

For cell immunofluorescent staining, cells were washed in PBS for three times and fixed with pure Acetone for 20 min at −20°C. Then, cells were blocked with serum solution and incubated with the primary antibodies overnight at 4°C. After rinsed again, immunostaining cells were incubated for 45 min at room temperature with Alexa Fluor 488- and 594-conjugated secondary antibodies and mounted with prolong gold antifade reagent with DAPI. 

### 2.7. Double Immunofluorescent Labeling

For a double immunofluorescence procedure, we incubated the sections and cells with two primary antibodies at 4°C overnight. We blocked samples with 10% donkey serum for 2 h at room temperature. The primary antibodies were anti-CD31 (1 : 200), anti-vWF (1 : 200), and anti-PDI (1 : 200). After washing in PBS, the sections and cells were incubated in the dark with a mixture of two secondary antibodies as described already.

### 2.8. Assessment of the Differentiation Capacity of Primo Vessel Cells *In Vitro*

The differentiation capacity of primo vessel cells was further tested *in vitro*. After cultured in DMEM with or without 5 ng/mL TGF-*β*1 (Pierce, Rockford, IL, USA) stimulation for indicated days, endothelial markers (vWF, CD31, VE-cadherin), and fibroblast markers (PDI, MTS1, procollagen I) expression in primo vessel cells were determined by immunocytochemistry. Culture media was refreshed every 3 days.

### 2.9. mRNA Isolation and Real-Time PCR

Total RNA was extracted from cultured primo vessel cells using the Trizol reagents according to the protocol provided by the manufacturer (Invitrogen, Carlsbad, CA, USA). The RNA content was measured spectrophotometrically. Then, total RNA (2 *μ*g) was treated with DNase I followed by synthesis of the first strand of DNA using reverse transcription system. Real-time PCR was performed in 25 *μ*L of reaction mixture containing 0.4 *μ*M primers, 2 *μ*L of cDNA product, 5 mM dNTPs, 0.75 units of Taq polymerase (Invitrogen, CA, USA), and 1 *μ*L of iQ-SYBR green supermix reagent (Bio-Rad, CA, USA). Fold changes of target gene mRNA levels relative to the endogenous glyceraldehyde-3-phosphate dehydrogenase (GAPDH) control were calculated. The sequences of the primers were listed in Table 1. The mRNA levels were expressed as fold changes after normalization.

### 2.10. ELISA Analysis

For collagen *α*1(I) secretion in primo vessel cells culture media, it was measured using ELISA method. Primo vessel cells were cultured in six-well plates at a density of 5 × 10^3^ cells/well for indicated days. Then, supernatants were collected, and a sandwich ELISA for collagen *α*1(I) (Santa Cruz Biotechnology, CA) was performed. Optical densities were measured using an ELISA plate reader at 450 nm. Results are expressed as fold increases of collagen *α*1(I) secretion compared with cells without TGF-*β*1 stimulation.

### 2.11. Statistics

All results were expressed as the means ± SE of at least three independent experiments. A one-way ANOVA was used to evaluate differences between groups (*P* < 0.05 was considered significant).

## 3. Results and Discussion

### 3.1. Identification of Primo Vessels in the Mesentery in Fresh Tissues and Cryosections

**Figure 1:**
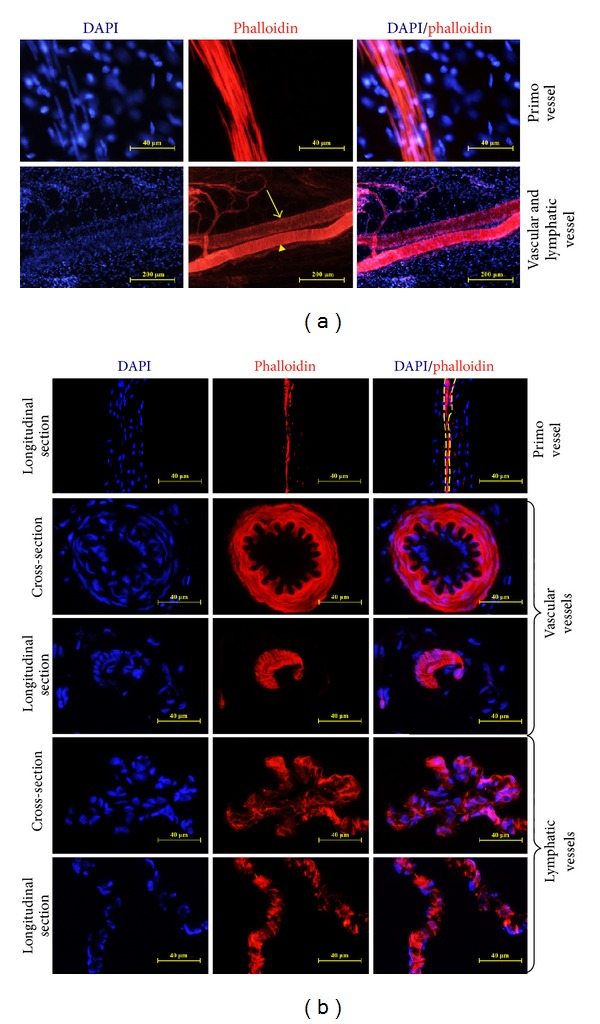
Primo vessels in the mesentery in fresh tissues and cryosections by phalloidin and DAPI staining. (a) The differences among primo vessels, vascular vessels, and lymphatic vessels. Primo vessels consist of phalloidin positive cells with characteristic rod-shaped nuclei, linear arrangements paralleling with the vessels. Vascular smooth muscle cells were also sensitive to phalloidin but showed thoroughly different appearances (yellow arrowheads). In lymphatic vessel, the endothelial cells formed the vessels in a network way (yellow arrows). (b) The differences among primo vessels, vascular vessels, and lymphatic vessels in cryosections. Phalloidin positive cells with rod-shaped nuclei; linear arrangements in longitudinal sections were primo vessels (yellow dashed area). Vascular vessels showed smooth muscular cells circulating the vessels and lymphatic vessels were formed in a network way.

Our previous studies have demonstrated that primo vessels located in the mesentery of rats [26]. They had prominent histological features such as rod-shaped nuclei with linear alignments, multiple-sinus, and collagen fibers inside the structures, and sometimes outer membranes were observed. We also found that using phalloidin (specific to F-actin) and DAPI staining, the cells' shape and arrangements of primo vessels were clearly detected. Here, we applied this method in distinguishing primo vessels from vascular vessels and lymphatic vessels. As shown in Figure 1(a), primo vessel cells had characteristic rod-shaped nuclei with linear arrangements, and their regular arrays along the vessels were clearly detected with phalloidin staining. Vascular smooth muscle cells were also sensitive to phalloidin but showed thoroughly different arrangement from primo vessels (Figure 1(a), arrowheads). As for lymphatic vessels, the endothelial cells were weakly positive to phalloidin and showed as a network structure (Figure 1(a), arrows). On the basis of phalloidin/DAPI double staining in reliably distinguishing primo vessels, vascular vessels, and lymphatic vessels in fresh tissues, we further employed this method in cryosections. As shown in Figure 1(b), in longitudinal cryosections, the structures with rod-shaped nuclei in linear alignments, and positive phalloidin expression (yellow dashed area) were considered as primo vessels. This method established in previous researches [23, 24, 26] was used in the current work as a time-saving method to detect primo vessels not only in tissue samples but also in longitudinal cryosections. 

### 3.2. Endothelial and Fibroblast Markers Expressed in Primo Vessels in the Mesentery

**Figure 2:**
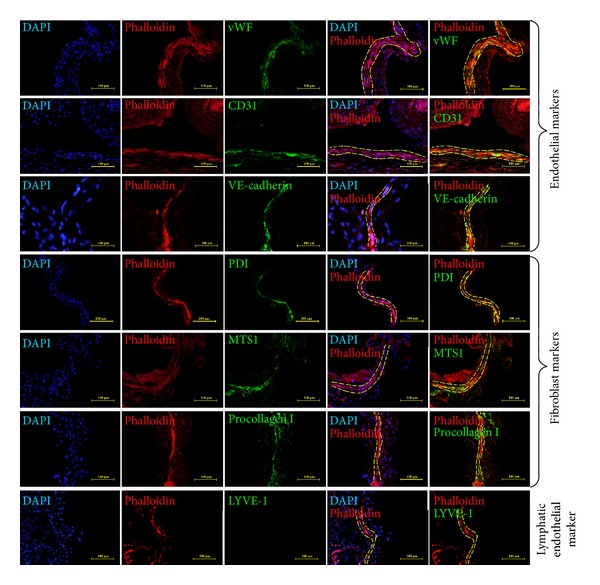
Immunofluorescent staining of primo vessels in the mesentery with endothelial markers, fibroblast markers, and phalloidin in longitudinal sections. Endothelial and fibroblast markers expressed in primo vessels in the mesentery. Antibodies to endothelial markers (vWF, CD31, VE-cadherin), fibroblast markers (PDI, MST1, procollagen I), and lymphatic endothelial marker (LYVE-1) (green) were used. DAPI (blue) and phalloidin (red) were used to identify primo vessels (yellow dashed area). Both of endothelial markers and fibroblast markers (green) were expressed in primo vessels. Lymphatic endothelial marker was negative in primo vessels.

**Figure 3:**
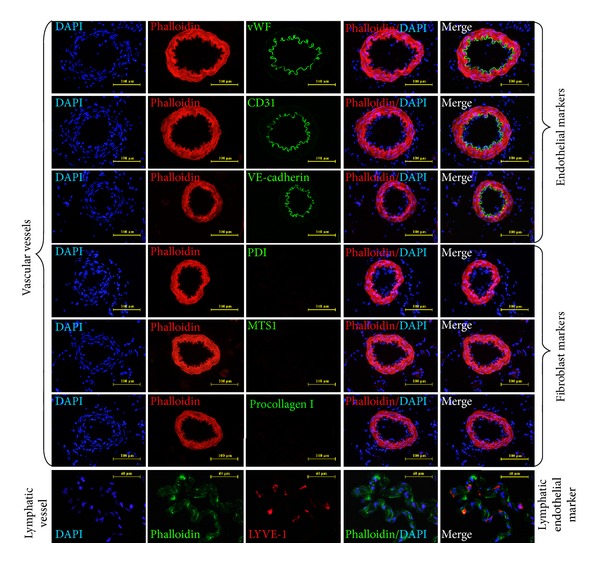
Controls for immunofluorescent staining of primo vessels in the mesentery. Vascular vessels were tested for endothelial (vWF, CD31, VE-cadherin) and fibroblast markers (PDI, MTS1, procollagen I). LYVE-1 was chosen to mark lymphatic endothelial cells.

**Figure 4:**
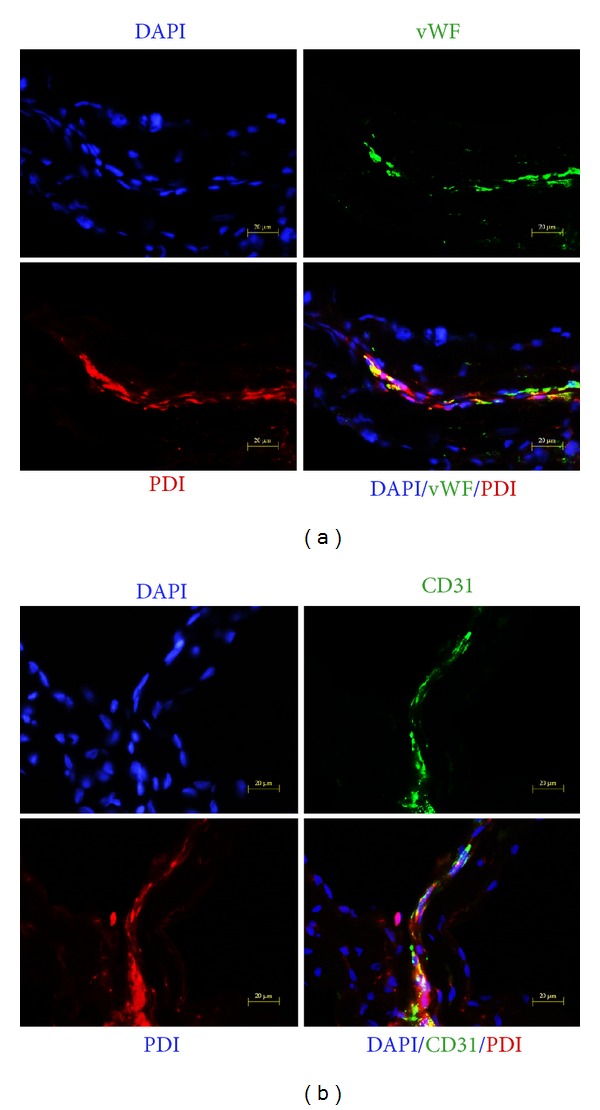
Double labeling of primo vessels in the mesentery with vWF/PDI and CD31/PDI. (a) Fluorescent microscopy of cryosections double-labeled with antibodies to vWF (green) and PDI (red). DAPI was used for labeling of nuclei (blue). In addition to the single-color panels, the pictures were merged. Primo vessel cells with rod-shaped nuclei and linear alignments were labeled positive for both vWF and PDI (yellow, white arrows). (b) CD31/PDI double labeling of primo vessels in the mesentery. Fluorescent microscopy of cryosections double-labeled with antibodies to CD31 (green) and PDI (red). DAPI was used for labeling of nuclei (blue). Accumulation of CD31/PDI positive cells (yellow, white arrows) correlated with areas of primo vessels with rod-shaped nuclei, linear alignments.

To investigate the biological properties of primo vessels, we first examined their endothelium features. Our results revealed that endothelial markers (vWF, CD31, VE-cadherin) were positive in primo vessels (Figure 2, green) and indicated their endothelium properties. Then, we performed fibroblasts markers (PDI, MTS1, procollagen I) in our experiments. As shown in Figure 2, primo vessels also positively expressed fibroblast markers (green). We concluded that primo vessels in the mesentery also possessed mesenchymal features. In addition, primo vessels negatively expressed a lymphatic endothelial cell marker, LYVE-1. As a control, endothelial markers (vWF, CD31, VE-cadherin) and fibroblast markers (PDI, MTS1, procollagen I) were also tested in vascular vessels (Figure 3). Next, we performed double-labeling experiments with endothelial and fibroblast markers to verify the possible properties of both endothelium and mesenchymal in primo vessels. The colocalization of vWF/PDI and CD31/PDI in primo vessels (Figure 4(a)) demonstrated that primo vessels are a transitional structure between endothelium and mesenchymal.

### 3.3. Endothelial and Fibroblast Markers Expressed in Primary Primo Vessel Cells

**Figure 5:**
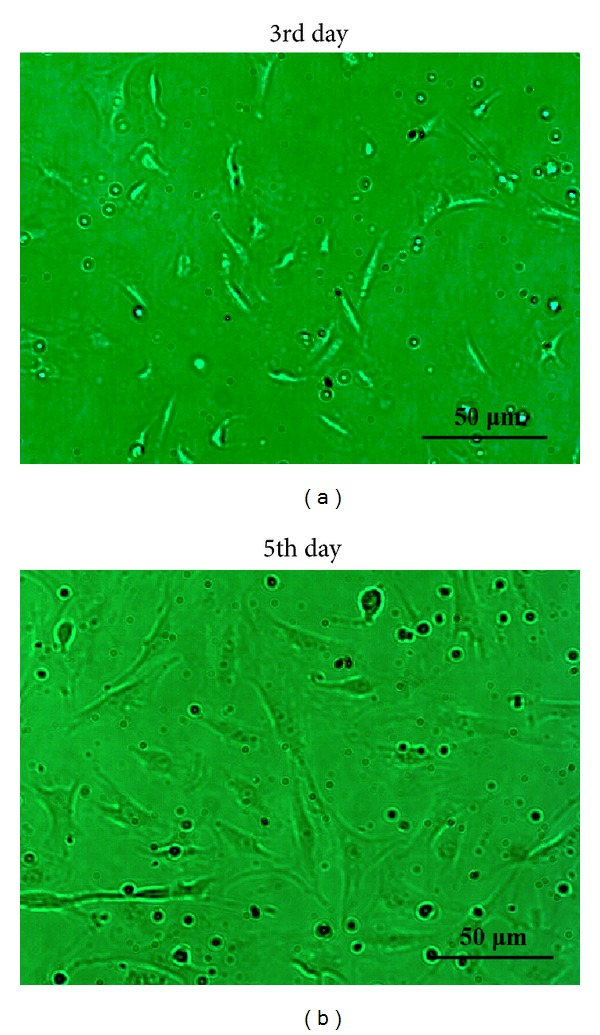
Primary primo vessel cell culture and identification. Primary primo vessel cells on 3rd and 5th day. Primo vessel cells started to spread on the 3rd day and showed a fibroblast-like phenotype on the 5th day.

**Figure 6:**
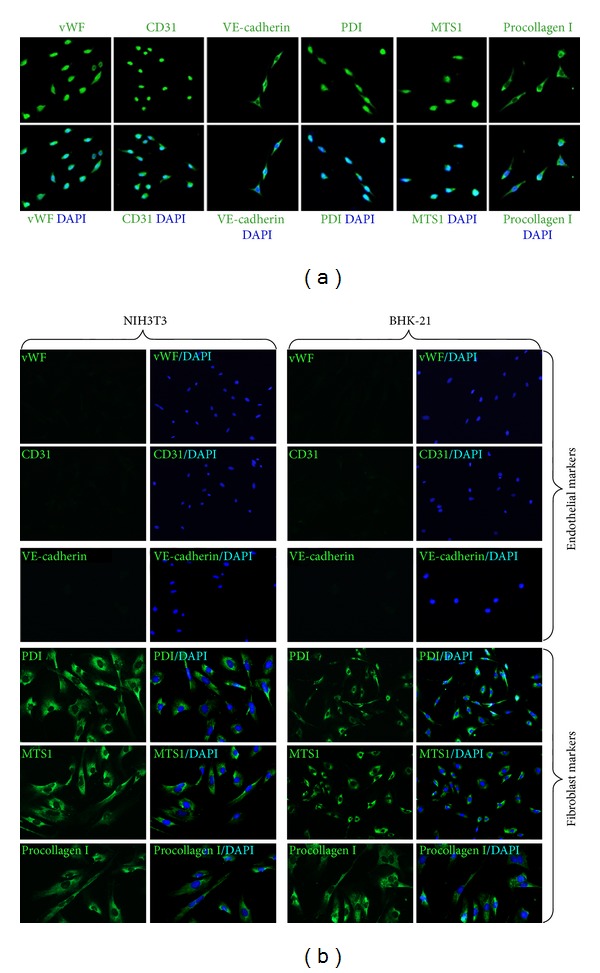
Immunofluorescent staining of primary primo vessel cells with endothelial markers and fibroblast markers. (a) Labeling of primo vessel cells with endothelial and fibroblast markers. Antibodies to endothelial markers (vWF, CD31, VE-cadherin) and fibroblast markers (PDI, MST1, procollagen I) (green) were used. Fluorescent microscopy revealed that both of endothelial markers and fibroblast markers (green) were expressed in primo vessel cells. (b) Controls for immunofluorescent staining of primo vessel cells. NIH3T3 cells and BHK-21 cells were positive to fibroblast markers (PDI, MTS1, procollagen I) (green) whereas negative to endothelial markers (vWF, CD31, VE-cadherin).

**Figure 7:**
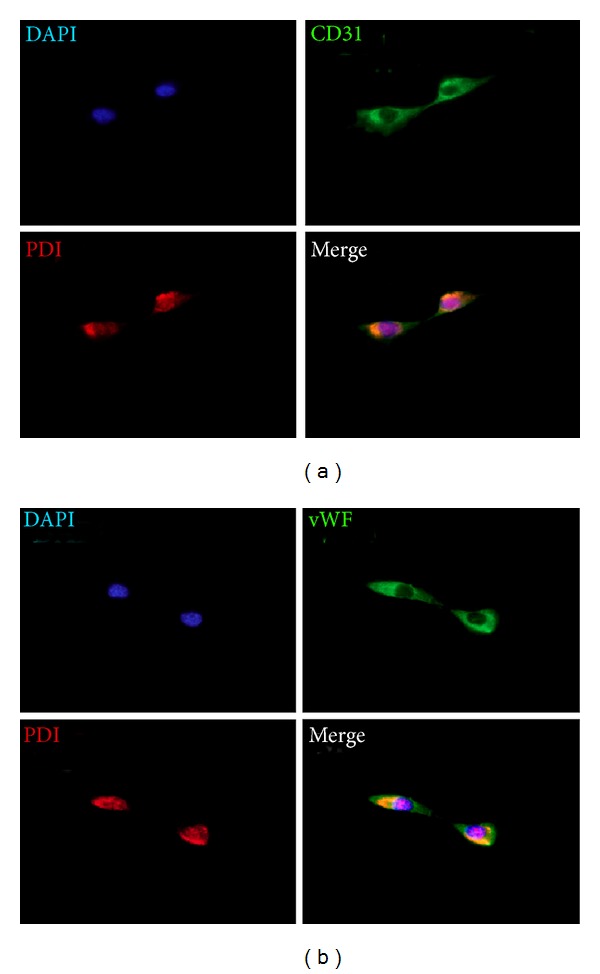
Double labeling of primary primo vessel cells with vWF/PDI, CD31/PDI. (a) Fluorescent microscopy of primo vessel cells double-labeled with antibodies to vWF (green) and PDI (red). DAPI was used for labeling of nuclei (blue). In addition to the single-color panels, the pictures were merged. Primo vessel cells were labeled positive for both vWF and PDI (orange). (b) CD31/PDI double labeling of primo vessel cells in the mesentery. Fluorescent microscopy of primo vessel cells double-labeled with antibodies to CD31 (green) and PDI (red). DAPI was used for labeling of nuclei (blue). Primary primo vessel cells coexpressed CD31/PDI (yellow).

To further demonstrate our observation of endothelial and fibroblast markers in primo vessels, we isolated and cultured primary primo vessel cells in rat mesentery. According to the potential biological features of fibroblasts in primo vessel cells, we performed collagenase digestion method in primary primo vessel cell isolation. Our data showed that on the 3rd and 5th day, primo vessel cells showed fibroblast-like shapes (Figure 5). We stained primo vessel cells with antibodies to endothelial markers (vWF, CD31, VE-cadherin) and fibroblast markers (PDI, MST1, procollagen I) (Figure 6(a)). The results revealed that all these markers positively expressed in primo vessel cells. NIH3T3 cell line and BHK-21 cell line were selected as controls and only positive to fibroblast markers (PDI, MST1, procollagen I) (Figure 6(b)). Double labeling with vWF/PDI and CD31/PDI supported the conception that primo vessel cells were ascribed to a phenotype between endothelial cells and fibroblasts (Figure 7). 

### 3.4. Differentiation of Primo Vessel Cells into Fibroblasts *In Vitro*

**Figure 8:**
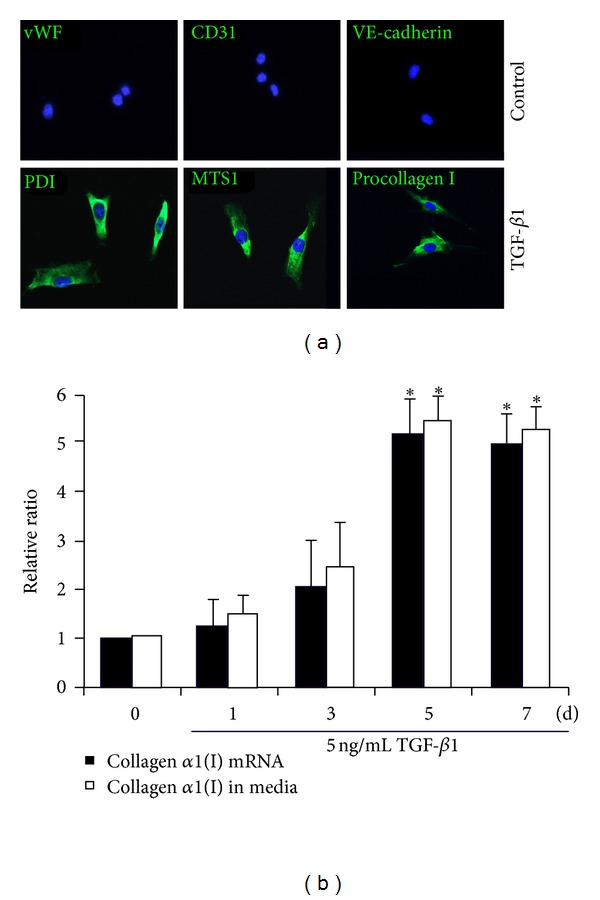
Assessment of differentiation of primo vessel cells *in vitro*. (a) Primary primo vessel cells were cultured in DMEM supplemented with or without 5 ng/mL TGF-*β*1 for 5 days, and then endothelial markers (vWF, CD31, VE-cadherin) and fibroblast markers (PDI, MTS1, procollagen I) were tested by immunocytochemistry. (b) Primary primo vessel cells were cultured in DMEM supplemented with or without 5 ng/mL TGF-*β*1 for indicated days collagen *α*1(I) mRNA was examined by real-time PCR, and collagen *α*1(I) secretion in culture media was detected by ELISA. *P < 0.01 versus TGF-*β*1-simulated primo vessel cells (the first column on the left in each group).

Our results indicated that in the mesentery, primo vessels were a unique transitional structure possessing both endothelial and mesenchymal properties. We deduced that primo vessel cells had differentiation potential. Under different stimulations, they might be induced and perform endothelial cells or fibroblasts functions. To verify this deduction, we studied the plasticity of primo vessel cells *in vitro*. We found that with the induction of TGF-*β*1, primo vessel cells transformed into fibroblasts over 5 days. Endothelial markers (vWF, CD31, VE-cadherin) diminished, and just fibroblast markers (PDI, MTS1, procollagen I) were positive in these cells (Figure 8(a)). One of the important functions of fibroblasts is to synthesize and produce extracellular matrix (ECM), especially collagen *α*1(I) [27, 28]. We found that in contrast to primo vessel cells, both the collagen *α*1(I) mRNA and protein secretion obviously increased in cells with TGF-*β*1 stimulation (Figure 8(b)). These data demonstrated that primo vessel cells differentiated into fibroblasts. Primo vessel cells are a new source of fibroblasts.

## 4. Conclusions

In conclusion, our research demonstrated that primo vessels in the mesentery were a special transitional structure between endothelium and mesenchymal. Primo vessel cells had the capability of differentiation into fibroblasts *in vitro*. It is a new finding of EndMT in normal postnatal states.

## Figures and Tables

**Table 1:** The sequences of the primers.

Primer	Sequences (5′-3′)	Size
Rat collagen *α*1(I)	Forward: TTCCCTGGACCTAAGGGTACT	113 bp
	Reverse: TTGAGCTCCAGCTTCGCC	
Rat GAPDH	Forward: TGGCCAAGGTCATCCATGAC	75 bp
	Reverse: GAGTGGCAGTGATGGCATGG	
